# Conditional Inhibition of Adult Neurogenesis by Inducible and Targeted Deletion of ERK5 MAP Kinase Is Not Associated with Anxiety/Depression-Like Behaviors^1^,^2^

**DOI:** 10.1523/ENEURO.0014-14.2015

**Published:** 2015-04-16

**Authors:** Junhui Zou, Wenbin Wang, Yung-Wei Pan, Glen M. Abel, Daniel R. Storm, Zhengui Xia

**Affiliations:** 1 Toxicology Program in the Department of Environmental and Occupational Health Sciences; 2 Department of Pharmacology, University of Washington, Seattle, Washington 98195

**Keywords:** anxiety, depression, ERK5, MAP kinase, neurogenesis

## Abstract

Although there is evidence that adult neurogenesis contributes to the therapeutic efficacy of chronic antidepressant treatment for anxiety and depression disorders, the role of adult neurogenesis in the onset of depression-related symptoms is still open to question. To address this issue, we utilized a transgenic mouse strain in which adult neurogenesis was specifically and conditionally impaired by Nestin-CreER-driven, inducible knockout (icKO) of *erk5* MAP kinase in Nestin-expressing neural progenitors of the adult mouse brain upon tamoxifen administration. Here, we report that inhibition of adult neurogenesis by this mechanism is not associated with an increase of the baseline anxiety or depression in non-stressed animals, nor does it increase the animal’s susceptibility to depression after chronic unpredictable stress treatment. Our findings indicate that impaired adult neurogenesis does not lead to anxiety or depression.

## Abstract (precis)

New neurons are continuously born in two regions of the adult mammalian brain, the hippocampus and the olfactory bulb, through a process called adult neurogenesis. These adult-born neurons are critical for learning and memory, as well as olfaction.

## Significance Statement

New neurons are continuously born in two regions of the adult mammalian brain, the hippocampus and the olfactory bulb, through a process called adult neurogenesis. These adult-born neurons are critical for learning and memory, as well as olfaction. Furthermore, an increase in adult neurogenesis likely contributes to the therapeutic efficacy of chronic antidepressants. However, whether impaired adult neurogenesis underlies the etiology of anxiety and depression is still unclear. This study aims to address this question by utilizing a genetic mouse model in which the production of adult-born neurons is inducibly and selectively impaired. Our data suggest that impaired adult neurogenesis alone does not contribute to anxiety or depression, nor does it increase depression after chronic stress.

## Introduction

Adult neurogenesis occurs in the subgranular zone of the dentate gyrus and the subventricular zone along the lateral ventricles in mammalian brains (Altman and Das, 1965; Alvarez-Buylla et al., 1988). Adult-born neurons in the dentate gyrus functionally integrate into the hippocampal circuitry and play a critical role in cognitive processes such as contextual and spatial memory and pattern separation. Interestingly, there is also a link between adult neurogenesis and psychiatric disorders including anxiety and depression (Kheirbek et al., 2012; Petrik et al., 2012). Sustained stress exposure not only induces maladaptive fear responses and depression, but it also decreases the production and survival of adult-born hippocampal neurons in animal models (Duman, 2004; Warner-Schmidt and Duman, 2006). Furthermore, treatment of anxiety/depression with antidepressants normalizes the level of adult neurogenesis (Czéh et al., 2001; David et al., 2009; Koo et al., 2010; Garza et al., 2011). Studies that combined depression animal models and neurogenesis-ablation animal models suggest that intact adult neurogenesis is critical for antidepressant-mediated reversal of the depressive state (Santarelli et al., 2003; David et al., 2009; Revest et al., 2009; Koo et al., 2010; Surget et al., 2011).

Despite these exciting discoveries, conflicting evidence exists regarding a direct link between impairment of adult neurogenesis and the etiology of depression/anxiety. For example, although a recent study supports a causal role for decreased adult neurogenesis in depression (Snyder et al., 2011), other studies in the literature report the absence of depression-like behavior in animals with impaired adult neurogenesis (Santarelli et al., 2003; Surget et al., 2008; David et al., 2009; Jayatissa et al., 2010; Surget et al., 2011; Iascone et al., 2013; Jedynak et al., 2014; Pascual-Brazo et al., 2014). Furthermore, stress does not always decrease adult neurogenesis (Reif et al., 2006; Lagace et al., 2010). The increase in adult neurogenesis caused by exercise is paradoxically associated with an increased level of anxiety (Fuss et al., 2010), and some effects of antidepressants are neurogenesis-independent (David et al., 2009; Mendez-David et al., 2013; Jedynak et al., 2014). There is a general consensus that adult-born neurons in the dentate gyrus contribute to efficacy of antidepressants in the treatment of anxiety and depression. However, the participation of adult hippocampal neurogenesis in the onset of anxiety and depression-related symptoms is still not clear. Whether adult neurogenesis is involved in the modulation of baseline anxiety and depression or in maintaining appropriate emotion and mood in the context of stress are key unanswered questions. Further studies using different neurogenesis-ablation strategies and multiple anxiety and depression animal models are needed to clarify this issue.

ERK5 is a MAP kinase whose expression in the adult brain under normal physiological conditions is limited to the neural stem/progenitor cells, transiently amplifying progenitors, and/or newborn neurons in the adult neurogenic regions; it is not expressed in mature neurons in the adult brain (Pan et al., 2012b; Pan et al., 2012c; Pan et al., 2012d; Li et al., 2013). Tamoxifen administration to adult *Nestin-CreER™/ERK5^loxP/loxP^* mice leads to inducible deletion of *erk5* in the adult brain (Pan et al., 2012b; Pan et al., 2012c; Pan et al., 2012d; Li et al., 2013). This deletion is specific to Nestin-expressing neural progenitors, occurs only after tamoxifen administration, and sustains for at least 3 months. Deletion of *erk5* disrupts adult neurogenesis in hippocampus as well as the subventricular zone, and impairs multiple forms of hippocampus-dependent learning and memory, including contextual fear memory, spatial learning and memory, and pattern separation (Pan et al., 2012c; Pan et al., 2012a, 2013).

To investigate whether the impairment of adult neurogenesis in ERK5 inducible knockout (*icKO*) mice is associated with anxiety and depression-like behaviors, we subjected the mice to a series of behavioral tests to evaluate their anxiety and depression. We report that inhibition of adult neurogenesis by *erk5* deletion does not induce anxiety or depression-like behaviors in non-stressed animals, nor does it increase an animal’s susceptibility to depression in the context of stress.

## Materials and Methods

### Animals

*Nestin-CreER*™ (Kuo et al., 2006) and *ERK5^loxP/loxP^* (Wang et al., 2005) mice were crossed to yield *Nestin-CreER™/ERK5^loxP/+^* animals. *Nestin-CreER™/ERK5^loxP/+^* mice were further crossed with *ERK5^loxP/loxP^* mice to yield homozygous *Nestin-CreER™/ERK5^loxP/loxP^* animals, which were used for experimental breeding. These mouse strains were the same as previously described (Pan et al., 2012a; Pan et al., 2012b; Pan et al., 2012c; Pan et al., 2012d; Li et al., 2013). All animal experiments were performed with identically treated and handled littermate controls. Because there may be sex differences in adult neurogenesis (Barha et al., 2011; Roughton et al., 2012; Hillerer et al., 2013) and there are generally more individual variations using female mice in behavior tests because of their short estrous cycles, we only used male mice for behavioral tests in this study to reduce variation and the number of animals needed. A small cohort of *Nestin-CreER™/ERK5^loxP/loxP^* mice were also bred with *Gt(ROSA)26Sor-YFP* (R26-YFP) mice (Srinivas et al., 2001) to yield *Nestin-CreER^TM^/ERK5^loxP/loxP^/R26-YFP^loxP/loxP^* mice (Li et al., 2013) for cellular studies. Unless specified, mice were group housed under standard conditions (12 h light/dark cycle) with food and water provided *ad libitum*. All animal procedures were performed according to the regulation of the authors’ university’s animal care committee.

### Administration of tamoxifen

**Figure 1:**
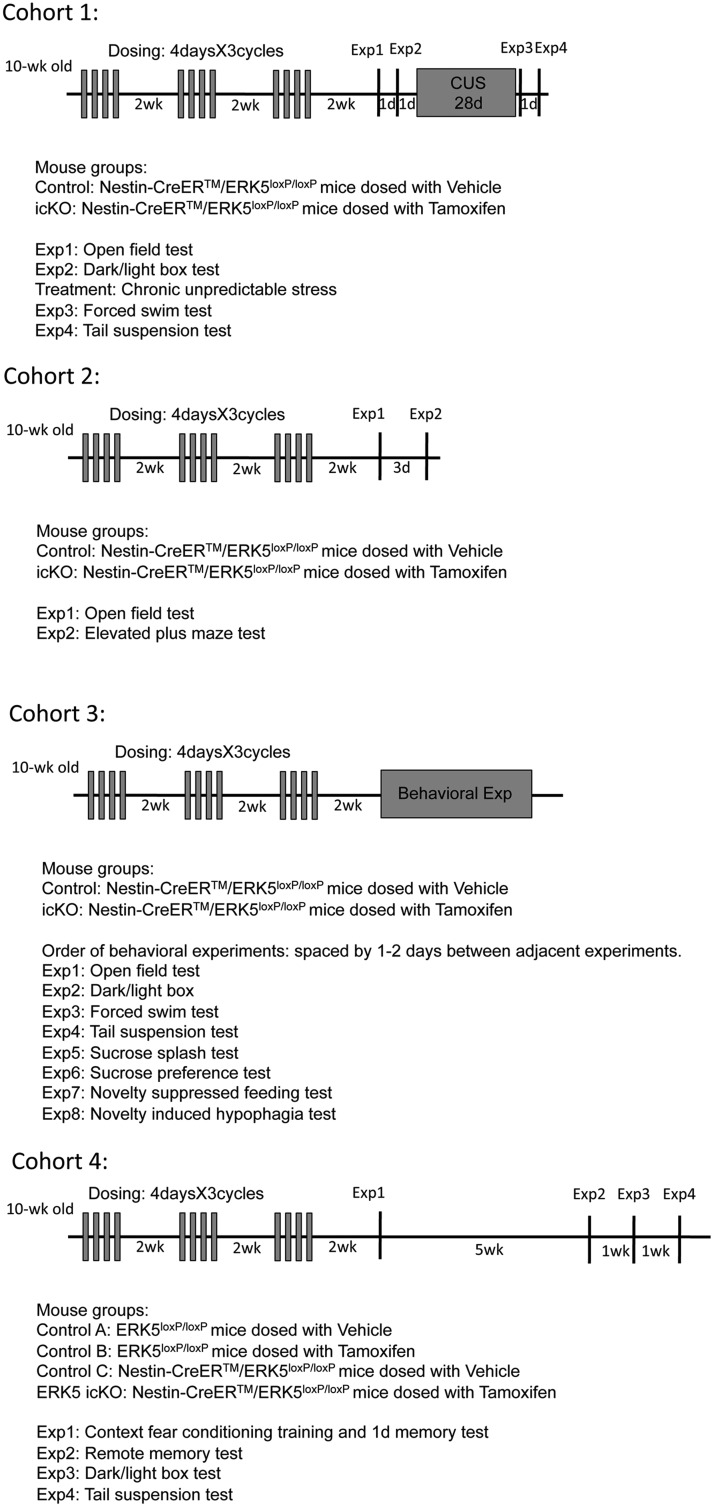
Mouse cohorts used for behavior tests and experimental timeline for each cohort.

To initiate Cre-mediated recombination, 10- to 12-week-old mice were dosed with 200 mg/kg tamoxifen or vehicle (corn oil with 2% acetic acid) via oral gavage once per day for 4 d per cycle, for a total of three cycles with a 2 week inter-cycle interval (3 × 4 d), exactly as previously described (Pan et al., 2012c; Pan et al., 2012d; Li et al., 2013; Wang et al., 2013). The tamoxifen administration to most of the animals (cohorts 2-4) was performed by the same investigator (Dr. Pan) as in previous studies (Pan et al., 2012a; Pan et al., 2012b; Pan et al., 2012c; Pan et al., 2012d). The tamoxifen-treated *Nestin-CreER™/ERK5^loxP/loxP^* mice were designated as the ERK5 inducible and conditional knock-out (*icKO*) mice, while vehicle-dosed littermates of the same genotype or tamoxifen-treated *ERK5^loxP/loxP^* littermates were used as controls for all behavioral experiments (see Fig. 1 for controls included in each cohort).

### Animal behavior tests

Four different cohorts of mice were used for behavior tests in this study (Fig. 1). A timeline for each cohort, showing the order of behavior tests and the length of time between each behavioral assay is illustrated in Figure 1. There was no statistically significant difference between the body weight of control and ERK5 *icKO* mice. For example, at the end of all behavioral tests for cohort 3 mice, the average body weight was 23.963 ±0.693 g (mean ± SEM) for control and 23.238 ± 0.466 g (mean ± SEM) for ERK5 *icKO*, *p* > 0.05. The light levels (lux) for each behavioral test are: open field: 250 lux; elevated-plus maze: 250 lux; dark/light box: light chamber: 300 lux; sucrose splash test: 250 lux; sucrose preference test: 250 lux; forced-swim test: 250 lux; tail suspension test: 250 lux; novelty-induced hypophagia: 1000 lux; novelty-suppressed feeding: 1000 lux.

### Open-field assay

The assay was performed 2 weeks after the last dose of tamoxifen. Mice were placed in an unfamiliar arena with clear side walls (10 × 10 × 16 inch; TruScan) and were allowed to freely explore the arena for 20 min. They were returned to their home cages after the test. Their locomotor activity was tracked by photo beams preinstalled to the arena and then analyzed by TruScan Software (Coulbourn Instruments).

### Elevated-plus maze

A beige elevated-plus maze apparatus was used for this experiment (San Diego Instruments). The maze consists of two perpendicular open arms, two perpendicular closed arms, and a center area. Each arm measures 50 × 5 cm and the center area measures 5 × 5 cm. The one-third distal portion of the two open arms and the two closed arms were defined as the open ends and the closed ends, respectively. The maze was placed in the center of a room with its stage 40 cm above the floor level and all the arms at least 50 cm away from any object in the room. Animals were placed in the center of the maze facing toward one of the open arms and allowed to freely explore the stage for 5 min. A video camera was installed on the ceiling of the room and directly above the center of the maze. The video camera was connected with a computer and ANY-maze software (San Diego Instruments) was used to track and analyze the movement in a real-time mode.

### Dark/light exploration assay

The test was conducted in a two-chamber shuttle box with an opaque divider in the middle (Coulbourn Instruments). Each chamber measures 17 × 17 × 33 cm. An opening measuring 6 × 6 cm was located at the center bottom of the divider. The walls of one chamber were made of black plexiglass while those of the other chamber were transparent. The ceiling of the box was made of a sheet of aluminum with a round hole measuring 1 cm in diameter over the center of the light chamber. Mice were habituated in the room for 1 h before the test with the room lights off. During the test, the room was not illuminated but the light compartment was lit by a 60W bulb closely placed over the hole on the ceiling. During the test, a mouse was first placed into the dark chamber and allowed to freely travel between the chambers for 5 min. The time spent in the light chamber was scored.

### Splash test

The splash test was performed by splashing a sucrose solution on the animal’s belly. Briefly, mice were split into individual cages and singly housed for at least 1 week prior to the test. On the day of testing, mice were splashed with a 10% sucrose solution on their belly using a wooden cotton-tipped applicator and quickly returned to the home cages. Grooming behavior, defined as touching, scrubbing, or licking the belly with snout, was monitored for 5 min. The latency to the first groom, the number of grooms, and total duration of the grooming were recorded by an experimenter blind to the genotype and treatment of the mice.

### Sucrose preference assay

Mice were given access to both plain water and a sucrose solution and their preference for the sucrose solution was quantified. Briefly, 3 d prior to the testing, singly housed mice were habituated to sucrose by being given a water bottle containing plain water and a second bottle with 1% sucrose with the left/right location balanced across animals and switched every day. After 3 d of habituation, both bottles were removed for 16 h overnight (5 P.M. to 9 A.M.). The water and sucrose bottles were then reintroduced in reversed left/right locations to the mice for 24 h. Bottles were weighed before the test, 20 min after the start of the test, and at the end of the test. The total drinking was expressed as the sum of the consumptions from both the plain water and sucrose bottles. The sucrose preference was expressed as the percentage of sucrose consumption of the total liquid consumption.

### Novelty-suppressed feeding test

The novelty-suppressed feeding test was performed by scoring the latency to feed for a food-deprived mouse when it is introduced to an unfamiliar environment. Briefly, prior to the test, individually housed mice were subjected to an 18 h (6 P.M. to 12 P.M.) food deprivation followed by food restriction (about 3 g of food per d) for 72 h. Mice were weighed before the food restriction and then weighed daily to avoid the loss of more than 15% of the body weight. Novelty-suppressed feeding assay was performed 20 h after the last feeding at 8 A.M. A hexagonal cylinder with clear Plexiglass base (hexagon of 12.5 cm for each side) and walls (height of 30 cm) was used as the test chamber. The cylinder was placed on a sheet of white filter paper to increase light reflection. A half pellet of mouse food (about 3 g) was placed on a piece of chemical weighing paper (3 × 3 cm) in the center of the floor of the cylinder. A mouse was removed from its home cage, weighed, and introduced to one corner of the cylinder. The mouse was allowed to freely investigate the cylinder and the food for a maximum of 15 min. Feeding behavior was observed by an experimenter blind to the genotype and treatment of the mouse. Once the mouse bit the food, the latency to biting was recorded and then the mouse and a new preweighed pellet of food were quickly transferred to the home cage. The mouse was undisturbed in the home cage for 5 min and then the food was removed and weighed again.

### Novelty-induced hypophagia assay

In novelty-induced hypophagia assay, mice were given access to chocolate milk in an unfamiliar environment and the latency to drink was scored. Briefly, singly housed mice were habituated to Darigold chocolate milk for 30 min per day for 3 consecutive days. On the fourth day, a mouse was introduced into a clean cage with the same dimensions as the home cage, and with access to a bottle of chocolate milk, but the cages had the following modifications to enhance aversiveness: no bedding, bright lighting, and white paper under the cage. The mouse was allowed to freely investigate the cage and drink chocolate milk for 15 min. The latency to drink the chocolate milk and the number of trips to the chocolate milk were scored by an experimenter blind to the genotype and treatment of the mice.

### Forced-swim assay

The test was performed by using a standard protocol of forced-swim test for mice. Briefly, a 4 L glass beaker (16.5 cm in diameter) filled with water (23-25 °C) to a depth of 17.8 cm was used as the apparatus. Mice were placed in the beaker and allowed to swim undisturbed for 6 min and then removed, dried, and returned to their home cages. Water was changed between each subject. The entire session was recorded from the side by a video camera. The video was scored later by an experimenter blind to the genotypes for the latency to first episode of immobility, the duration of immobility, and immobile episodes during the last 4 min of the session.

### Tail-suspension test

Tail-suspension test was performed by suspending a mouse from a horizontal bar 50 cm above the floor by adhesive tape placed approximately 1 cm from the tip of the tail. Immobility, including the latency to first immobile episode, the number of immobile episodes, and the accumulated time spent immobile, within a 6 min session was scored by an experimenter blind to the genotype and treatment of the mice.

### Chronic unpredictable stress treatment

Singly housed mice were treated with a 14 d schedule of chronic unpredictable stress (CUS) treatment for a total period of 4 weeks (2 repeats of the same 14 d schedule). The stressors included restraint (1.5 h), cold swim (15 °C, 10 min), wet bedding/cage tilt (45°, 16 or 20 h), inversion of the light/dark cycle (16 h), tail pinch (with plastic clothespins, 10 min), warm swim (25 °C, 6 min), inescapable shock (0.7 mA, 0.5 s/min ×3 min or 3 s/min ×3 min), and food and water deprivation (16 h) on a randomized schedule.

### Statistical analysis

All data were expressed as mean ± SEM. Comparison between the control and ERK5 *icKO* groups was analyzed by Student’s *t* test, two-tailed analysis. *n* = 9-12 for control group and 10-14 for ERK5 *icKO* mice in each test.

## Results

### Inhibition of adult neurogenesis by targeted deletion of ERK5 is not associated with an increased level of anxiety in non-stressed mice

**Figure 2:**
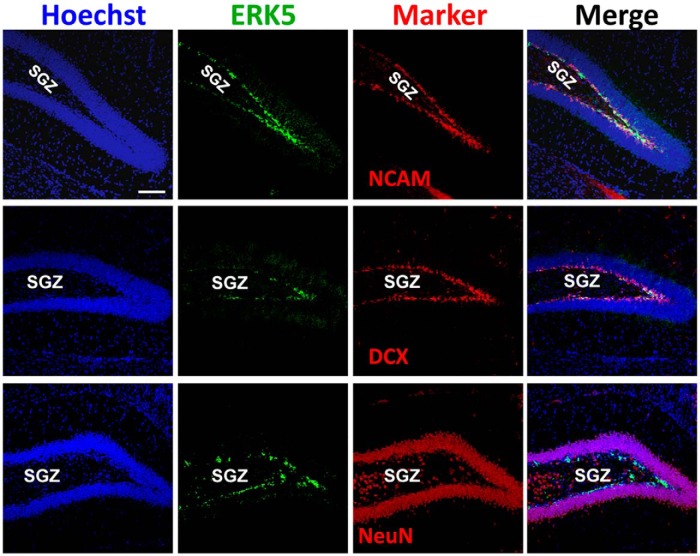
ERK5 MAPK expression in the dentate gyrus of the adult mouse brain. Images are representative immunostaining of coronal sections of adult mouse brain tissue showing ERK5 protein expression (green) primarily in transiently amplifying progenitors and/or newborn neurons (doublecortin^+^ (DCX) or NCAM^+^) but not in mature neurons (NeuN, red) in the SGZ. Hoechst staining (blue) was used to identify all cell nuclei. Scale bar represents 100 μm and applies to all panels.

Under normal conditions, ERK5 expression in the adult brain is limited to the neural stem/progenitor cells, transiently amplifying progenitors, and/or newborn neurons in the adult neurogenic regions including the subgranular zone (SGZ) of the dentate gyrus in the hippocampus and the subventricular zone/rostral migratory stream. ERK5 is not expressed in mature neurons (NeuN^+^) in the adult brain (Pan et al., 2012c; Pan et al., 2012d; Li et al., 2013; Wang et al., 2013). Data in Figure 2 confirm that ERK5 protein is specifically expressed along the SGZ of the dentate gyrus, and that it is colabeled with markers for transiently amplifying progenitors and/or newborn neurons (PSA-NCAM, DCX), but not with NeuN, a marker expressed in mature neurons.

**Figure 3:**
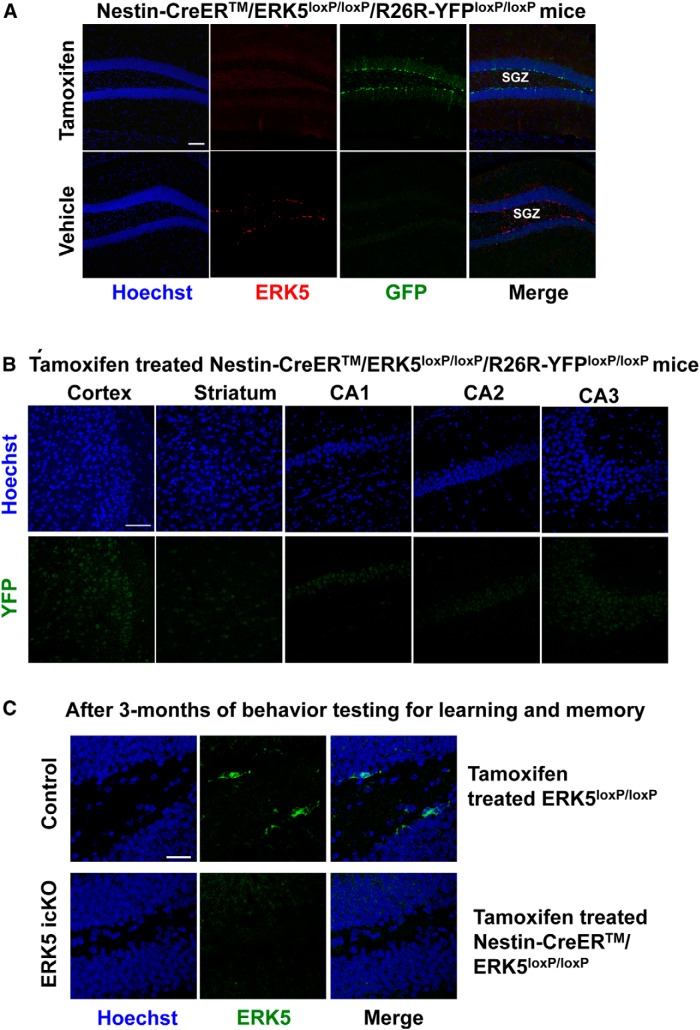
Characterization of the specificity of Nestin-CreER-mediated recombination in *ERK5 icKO* mice. ***A***, ERK5 and YFP immunostaining in the dentate gyrus of *Nestin-CreER^TM^/ERK5^loxP/loxP^/R26-YFP^loxP/loxP^* animals treated with tamoxifen or vehicle control, demonstrating the specificity and effectiveness of Nestin-Cre-ER^TM^-mediated *erk5* deletion. Scale bar, 100 μm. ***B***, YFP immunostaining in the cortex, striatum, and CA1-3 of the hippocampus of *Nestin-CreER^TM^/ERK5^loxP/loxP^/R26-YFP^loxP/loxP^* animals treated with tamoxifen, demonstrating that Nestin-Cre-ER^TM^-mediated recombination does not occur in non-neurogenic regions. Scale bar, 100 μm. ***C***, ERK5 immunostaining in the dentate gyrus of *ERK5 icKO* and control animals 3 months after behavior testing for learning and memory, demonstrating the sustained deletion of *erk5* during the time frames of behavior testing. Scale bar, 25 μm.

We utilized an inducible and conditional ERK5 knock-out (*ERK5 icKO*) mouse model to determine whether the inhibition of adult neurogenesis by ERK5 deletion alters baseline anxiety. In this model, adult *Nestin-CreER™/ERK5^loxP/loxP^* mice were treated with tamoxifen to induce *erk5* gene deletion in the Nestin-expressing neural stem cells (*ERK5 icKO*), while vehicle-treated mice of the same genotype or *ERK5^loxP/loxP^* littermates treated with tamoxifen or vehicle were used as controls (see Fig. 1 for details). Previous studies showed that Nestin-CreER™-driven *erk5* deletion occurs only upon tamoxifen administration (Pan et al., 2012c; Pan et al., 2012d; Li et al., 2013; Wang et al., 2013). To further examine the specificity of tamoxifen-induced, Nestin-CreER^TM^-mediated recombination, *Nestin-CreER^TM^/ERK5^loxP/loxP^* mice were crossed with *R26-YFP* reporter mice where Cre-mediated recombination removes a transcriptional STOP to allow YFP expression (Srinivas et al., 2001). *Nestin-CreER^TM^/ERK5^loxP/loxP^/R26-YFP^loxP/loxP^* mice were euthanized 10 d after the last dose of tamoxifen or vehicle control. There was ERK5 but no YFP expression in the SGZ of vehicle control treated mouse brain (Fig. 3*A*). In contrast, there were abundant YFP^+^ cells but no ERK5^+^ cells along the SGZ of tamoxifen-treated mouse brains. Furthermore, there were no YFP^+^ cells in the cortex, stratum, CA1, CA2, or CA3 of the hippocampus after tamoxifen treatment (Fig. 3*B*). These data indicate that *Nestin-Cre-ER^TM^*-mediated deletion of *erk5* is specific to adult neurogenic regions and there is no discernible deletion of *erk5* without tamoxifen or in non-neurogenic regions after tamoxifen treatment.

Deletion of *erk5* leads to impaired hippocampal adult neurogenesis primarily through delayed neuronal differentiation (Pan et al., 2012c; Pan et al., 2012d). The reduction of ERK5^+^ cells (75-80%) in the SGZ was similar at 3-4 weeks and 12 weeks after the last dose of tamoxifen treatment in naïve mice without behavior testing (Pan et al., 2012c; Pan et al., 2012d). Here we show that ERK5 protein is still not expressed in *ERK5 icKO* mice even after animals have been subjected to a series of behavior tests for learning and memory in a 3 month period (Fig. 3*C*).

**Figure 4:**
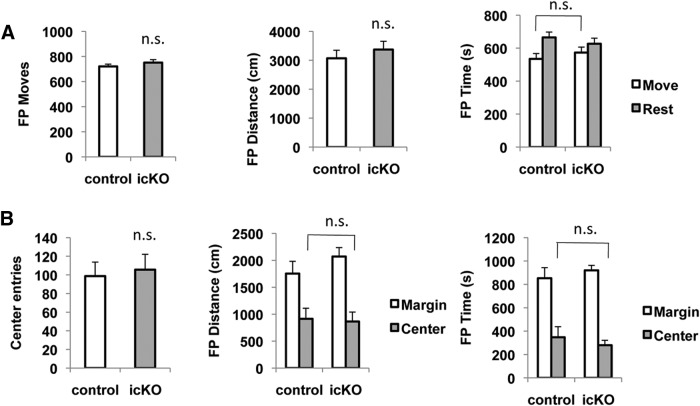
*ERK5 icKO* mice are not deficient in an open-field test. Naïve mice were introduced into an open-field arena for 20 min. Activity was scored by the TruScan software. ***A***, There was no difference in the total number of moves, the total distance traveled, or the total time spent moving in the floor plane between *ERK5 icKO* mice and control mice. ***B***, The *ERK5 icKO* mice did not exhibit reduced exploration in the center of the arena, shown as the number of entries to the center, the distance traveled, or the time spent in the center of the arena. n.s., Not significant.

Conditional deletion of *erk5* impairs multiple forms of hippocampus-dependent learning and memory, including contextual fear memory, spatial learning and memory, and pattern separation (Pan et al., 2012c; Pan et al., 2012a, 2013). To evaluate the level of anxiety of *ERK5 icKO* mice, we first subjected the mice to a series of behavior assays, including the open-field, elevated-plus maze, and the dark/light exploration tests, to test the animal’s adaptation and spontaneous exploration in new environments. In the open-field assay, a mouse was placed in an unfamiliar arena and locomotor activity was recorded. The level of anxiety is primarily assessed by the level of ambulation and the avoidance of exploration to the center of the arena (Bourin et al., 2007; Ramos, 2008). In comparison to control littermates, *ERK5 icKO* mice did not change the overall ambulation level, quantified as the number of moves (*p* = 0.324; Table 1)^1^, moving distance (*p* = 0.460)^2^, and moving time (*p* = 0.419)^3^ on the floor plane (Fig. 4*A*), nor did they show reduced exploration to the center of the arena, expressed as the number of entries to the center (*p* = 0.760)^4^, and the time spent in the center of the arena (*p* = 0.512)^5^ (Fig. 4*B*). These data indicate that non-stressed *ERK5 icKO* mice do not display overt anxiety-like behavior in the open-field assay.

**Table 1:** Statistical table

	Data structure	Type of test	Power
1*	Normal distribution	Two-tailed Student’s *t* test	15%
2	Normal distribution	Two-tailed Student’s *t* test	11%
3	Normal distribution	Two-tailed Student’s *t* test	12%
4	Normal distribution	Two-tailed Student’s *t* test	5%
5	Normal distribution	Two-tailed Student’s *t* test	20%
6	Normal distribution	Two-tailed Student’s *t* test	10%
7	Normal distribution	Two-tailed Student’s *t* test	10%
8	Normal distribution	Two-tailed Student’s *t* test	10%
9	Normal distribution	Two-tailed Student’s *t* test	11%
10	Normal distribution	Two-tailed Student’s *t* test	12%
11	Normal distribution	Two-tailed Student’s *t* test	3%
12	Normal distribution	Two-tailed Student’s *t* test	5%
13	Normal distribution	Two-tailed Student’s *t* test	25%
14	Normal distribution	Two-tailed Student’s *t* test	7%
15	Normal distribution	Two-tailed Student’s *t* test	3%
16	Normal distribution	Two-tailed Student’s *t* test	11%
17	Normal distribution	Two-tailed Student’s *t* test	23%
18	Normal distribution	Two-tailed Student’s *t* test	3%
19	Normal distribution	Two-tailed Student’s *t* test	13%
20	Normal distribution	Two-tailed Student’s *t* test	4%
21	Normal distribution	Two-tailed Student’s *t* test	72%
22	Normal distribution	Two-tailed Student’s *t* test	9%
23	Normal distribution	Two-tailed Student’s *t* test	35%
24	Normal distribution	Two-tailed Student’s *t* test	9%
25	Normal distribution	Two-tailed Student’s *t* test	26%
26	Normal distribution	Two-tailed Student’s *t* test	3%
27	Normal distribution	Two-tailed Student’s *t* test	12%
28	Normal distribution	Two-tailed Student’s *t* test	4%
29	Normal distribution	Two-tailed Student’s *t* test	11%
30	Normal distribution	Two-tailed Student’s *t* test	11%
31	Normal distribution	Two-tailed Student’s *t* test	5%
32	Normal distribution	Two-tailed Student’s *t* test	8%
33	Normal distribution	Two-tailed Student’s *t* test	6%
34	Normal distribution	Two-tailed Student’s *t* test	100%
35	Normal distribution	Two-tailed Student’s *t* test	80%
36	Normal distribution	Two-tailed Student’s *t* test	100%
37	Normal distribution	Two-tailed Student’s *t* test	28%
38	Normal distribution	Two-tailed Student’s *t* test	3%
39	Normal distribution	Two-tailed Student’s *t* test	10%
40	Normal distribution	Two-tailed Student’s *t* test	4%
41	Normal distribution	Two-tailed Student’s *t* test	3%
42	Normal distribution	Two-tailed Student’s *t* test	3%
43	Normal distribution	Two-tailed Student’s *t* test	8%
44	Normal distribution	Two-tailed Student’s *t* test	5%
45	Normal distribution	Two-tailed Student’s *t* test	5%
46	Normal distribution	Two-tailed Student’s *t* test	6%
47	Normal distribution	Two-tailed Student’s *t* test	6%
48	Normal distribution	Two-tailed Student’s *t* test	8%

*Numbers in this column refer to the corresponding superscripted numbers following *p* values in Results.

**Figure 5:**
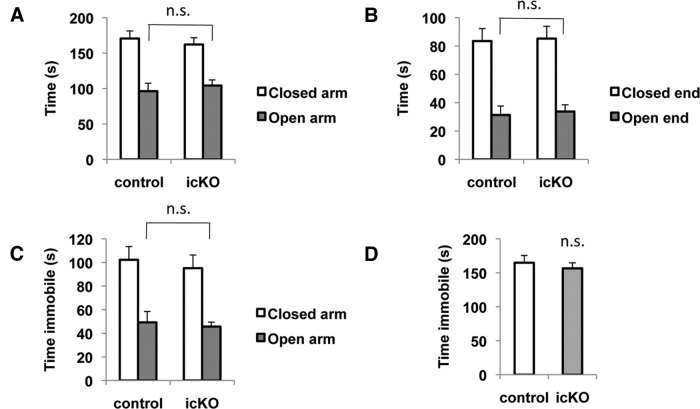
*ERK5 icKO* mice performed similarly to the controls in the elevated-plus maze. Mice were placed in the elevated-plus maze and allowed to explore for 5 min. Exploratory activity and immobility were analyzed by AnyMaze Software. ***A***, Time spent in open arms and closed arms. ***B***, Time spent in open and closed ends (the distal one-third of the open and closed arms). ***C***, Time spent immobile in the open and closed arms. ***D***, Total time immobile in the entire maze. n.s., Not significant.

To confirm the findings from the open-field assay, we subjected the *ERK5 icKO* and control mice to two approach−avoidance conflict-based behavioral paradigms for assessing anxiety behaviors: the elevated-plus maze and the light/dark exploration test. The elevated-plus maze is based on the natural aversion of rodents for open spaces; decreased exploration in the open arms is a sign of anxiety (Bourin et al., 2007; Ramos, 2008). The *ERK5 icKO* mice did not exhibit an increased avoidance to the open spaces compared to control mice, quantified by the amount of time spent in the open arms (*p* = 0.630)^6^ and the open ends defined as the distal one-third of open arms (*p * =0.758)^7^ (Fig. 5*A*,*B*). However, it is still possible that *ERK5 icKO* mice spent more time in immobility while they were in the open arms, a hidden sign of elevated maladaption. We therefore analyzed the immobile behavior in the open arms and found that *ERK5 icKO* mice did not exhibit an increase of immobility in the open arms (*p* = 0.725)^8^ (Fig. 5*C*), confirming that they were actively exploring the open arms as control mice did. Their overall fear response to a novel environment was not compromised by ERK5 deletion, as both groups of mice spent about 50% of time (150 s during the 5 min test) in immobility on the apparatus (*p* = 0.543)^9^ (Fig. 5*D*). These results indicate that *ERK5 icKO* mice are not deficient in their ability to explore an unfamiliar and open environment, and they do not exhibit increased anxiety in the elevated-plus maze test.

**Figure 6:**
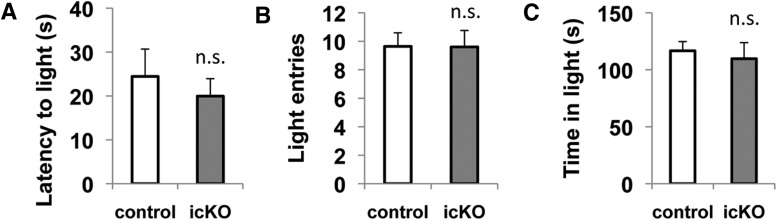
*ERK5 icKO* mice are not deficient in a dark/light exploration test. Mice were first placed into the dark compartment of a shuttle box with one dark and one light compartment. They were allowed to freely explore the box for 5 min. ***A***, The latency to the first entry of the light compartment. ***B***, The total entries to the light compartment. ***C***, The time spent in the light compartment. n.s., Not significant.

In contrast to the elevated-plus maze test, the light/dark exploration assay is based on the innate aversion of rodents to brightly illuminated areas. Mice were introduced into a novel shuttle box with one dark and one brightly illuminated compartment. Decreased tendency to explore the light compartment indicates elevated anxiety (Crawley and Goodwin, 1980; Bourin and Hascoët, 2003). The vehicle-treated control mice explored the light compartment substantially, although they still exhibited a preference for the dark compartment (2 min in light and 3 min in dark compartment over a 5 min period), consistent with the behavior of normal mice reported in the literature (Bourin and Hascoët, 2003). *ERK5 icKO* mice did not show a decreased tendency to explore the light compartment: the latency to their first entry (*p* = 0.553)^10^, total number of entries (*p* = 0.981)^11^, and accumulated time spent exploring the light compartment (*p* = 0.673)^12^ were not significantly different from their control littermates (Fig. 6). These data indicate that *ERK5 icKO* mice are competent in exploring an unfamiliar and brightly lit environment and do not exhibit anxiety in the dark/light exploration assay.

**Figure 7:**
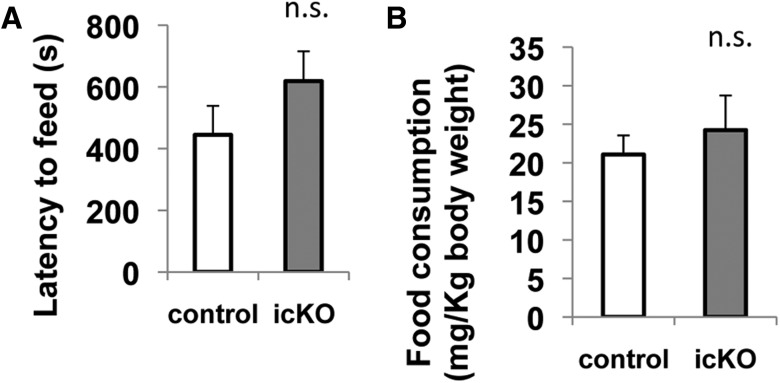
*ERK5 icKO* mice do not display enhanced hypophagia behavior in a novelty-suppressed feeding test. After 4 d of food restriction, individually housed mice were introduced into an unfamiliar environment with one pellet of regular mouse food. ***A***, Latency to eat the food pellet in the unfamiliar environment. ***B***, Food consumption in the home cage. Once the mouse started eating the food or the 15 min time limit was reached, the mouse and a new preweighed food pellet were quickly transferred to the home cage and the food consumption in the next 5 min was measured. n.s., Not significant.

Feeding in a new environment is an indication of adaptation, whereas the inhibition or delay of feeding behavior in a new environment, hypophagia, is a sign of anxiety (Dulawa and Hen, 2005). One commonly used hypophagia test is novelty-suppressed feeding, in which a food-deprived mouse is introduced to a new environment with a pellet of food and the latency to feed is quantified. Compared with the vehicle control mice, *ERK5 icKO* mice did not delay their first attempt to eat food (*p* = 0.212)^13^ (Fig. 7*A*). However, a confounding factor for this test is the animal’s degree of hunger and level of appetite, which may affect their motivation for food, thus the latency to feed. Therefore, food consumption in a familiar environment, in their home cages, to which they were returned after the test, was used to assess their motivation for food. There was no significant difference between *ERK5 icKO* mice and the control mice in their home cage food consumption (*p* = 0.545)^14^ (Fig. 7*B*). Together, these data suggest that *ERK5 icKO* mice do not display an elevated level of hypophagia, indicating that they do not have an increased level of anxiety in the novelty-suppressed feeding test.

**Figure 8:**
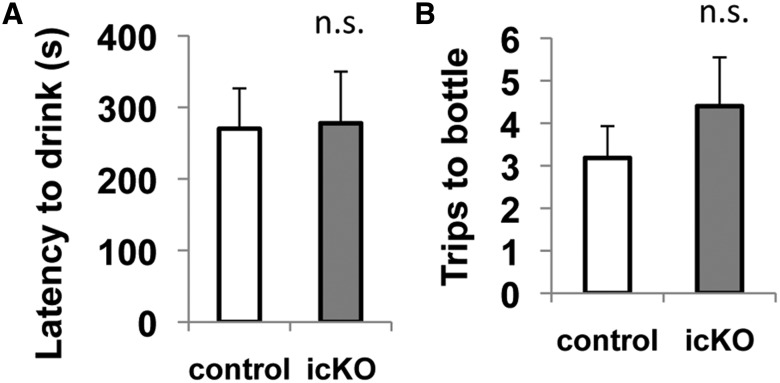
ERK5 icKO mice behave the same as control mice in a novelty-induced hypophagia test. Individually housed mice were trained with chocolate milk 30 min per day on 3 consecutive days in their home cages. On the fourth day, each mouse was transferred into a new mouse cage without bedding but with the access to a bottle of chocolate milk. The mouse was allowed to drink the milk freely for 15 min. ***A***, Latency to drink the chocolate milk. ***B***, Total number of trips to the chocolate milk. n.s., Not significant.

Mice were also subjected to another behavior test for hypophagia, the novelty-induced hypophagia test (Dulawa and Hen, 2005), in which a palatable snack (chocolate milk) was used and no food restriction is required. The test was performed in a new mouse cage without bedding, an unfamiliar but less aversive environment. Again, there was no difference between *ERK5 icKO* mice and control mice in the latency to drink the chocolate milk (*p* = 0.935)^15^ or the frequency of drinking (*p* = 0.387)^16^ within the observation period, confirming that ERK5 deletion does not cause hypophagia (Fig. 8).

### Impaired adult neurogenesis by targeted deletion of ERK5 is not associated with depression-like behaviors in non-stressed mice

Symptoms of depression include a lack of self-care, loss of interest in pleasure, and the feeling of hopelessness. An array of behavioral tests have been developed to assess each of these aspects of depression in rodents. For example, grooming behavior observed in the splash test is used to assess self-care, the sucrose-preference test is designed to evaluate the level of interest in pleasure, while the tail-suspension test and forced-swim test are two frequently used tests for quantification of hopelessness and despair.

**Figure 9:**
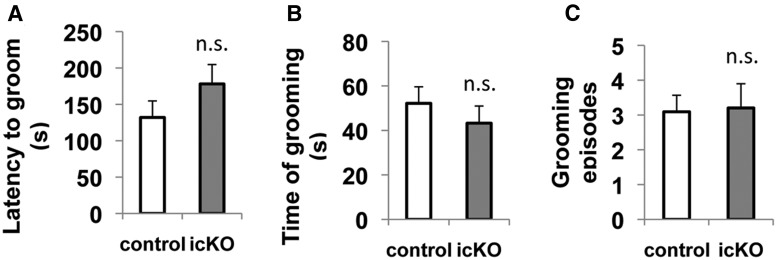
*ERK5 icKO* mice do not display reduced grooming behavior in the splash test. Mice were splashed with 10% sucrose solution on the abdomen and their grooming behavior was observed in the home cage for 5 min. ***A***, Latency to first groom. ***B***, Total time spent grooming. ***C***, Number of grooming episodes. n.s., Not significant.

To assess whether the inhibition of adult neurogenesis by ERK5 deletion is associated with depression-like behavior, we first evaluated self-care, specifically the grooming behavior, of the *ERK5 icKO* mice and control mice using the splash test. Mice were splashed with sucrose solution on their abdomen and their grooming behavior was then scored. We did not observe a difference between the two groups of mice in their latency to groom (*p* = 0.205)^17^, grooming frequency (*p* = 0.899)^18^, or the duration of grooming (*p* = 0.415)^19^ (Fig. 9), suggesting that inhibition of adult neurogenesis by ERK5 deletion does not affect self-care in non-stressed animals.

**Figure 10:**
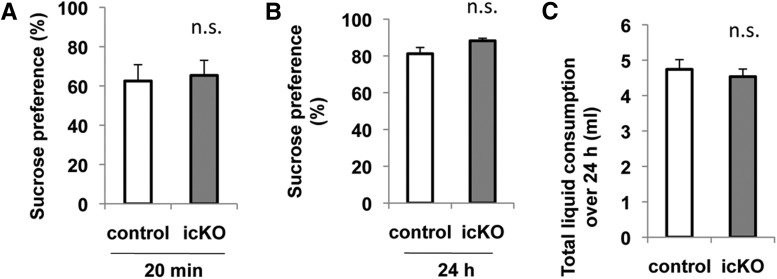
*ERK5 icKO* mice do not show more anxiety in the sucrose preference test. Mice were habituated to freely accessible plain drinking water and 1% sucrose water for 3 d. Then, after 16 h of water deprivation, they were given access to both plain and sucrose water for 24 h. The consumption from each bottle was measured after the first 20 min of the test and again at the end of the test. ***A***, Percentage of sucrose water consumption at 20 min. ***B***, Percentage of sucrose water consumption at the end of the 24 h test. ***C***, Total consumption from the two bottles in 24 h. n.s., Not significant.

Next, we evaluated whether ERK5 deletion affects the animal’s interest in pleasure using the sucrose-preference assay. Mice were given access to both sucrose water and plain water. A significant preference to sucrose water versus plain water indicates the interest for pleasure. We first habituated both the *ERK5 icKO* mice and control mice to freely available water and 1% sucrose for 3 d. After 16 h of fluid deprivation, mice were reintroduced to both bottles and their preference for each bottle was measured during a 20 min test followed by a 24 h test. Both groups preferred the sucrose drink over the plain water in the first 20 min (*p* = 0.799, *ERK5 icKO* mice vs control mice)^20^ (Fig. 10*A*) as well as over the next 24 h (*p* = 0.188, *ERK5 icKO* mice vs control mice)^21^ (Fig. 10*B*), and they consumed the same amount of total liquid (*p* = 0.468)^22^ (Fig. 10*C*).

**Figure 11:**
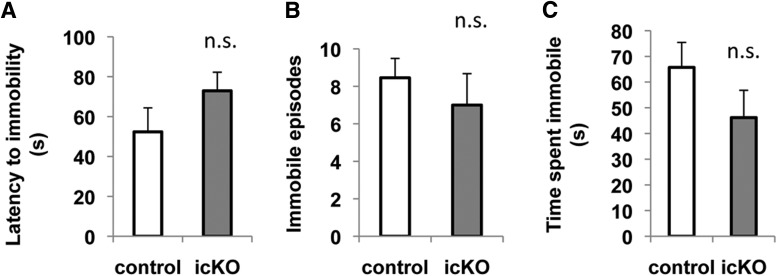
*ERK5 icKO* mice do not display increased despair behavior in a forced swim test. A 4 L glass beaker with water added to 17 cm from the base was used as the apparatus for this test. A mouse was dropped into the water and immobility was observed from the side for a total of 6 min. ***A***, Latency to the first episode of immobility. ***B***, Total number of immobile episodes in the last 4 min of the test. ***C***, Accumulated duration of immobility in the last 4 min of the test. n.s., Not significant.

**Figure 12:**
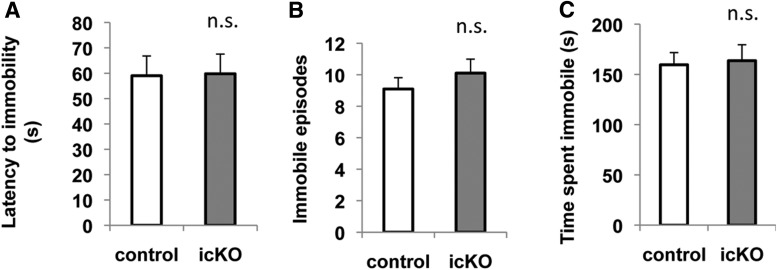
*ERK5 icKO* mice do not show increased despair behavior in a tail-suspension test. ***A***, Latency to the first episode of immobility. ***B***, Total number of episodes of immobility. ***C***, Accumulated duration of immobility. n.s., Not significant.

To assess hopelessness, we subjected *ERK5 icKO* mice and control mice to the forced-swim and tail-suspension tests. In the forced-swim test, a mouse is placed into an inescapable container filled with water. After a brief period of vigorous swimming, the mouse exhibits a characteristic immobile posture. Early onset of immobility and an increased level of immobility indicate increased behavioral despair or lowered mood (Porsolt et al., 1977). Compared with controls, *ERK5 icKO* mice showed no signs of increased despair in the forced-swim test, determined by the latency to the first immobile episode (*p* = 0.192)^23^, the immobility frequency (*p* = 0.470)^24^, and the total time spent immobile (*p* = 0.191)^25^ (Fig. 11). In the tail-suspension test, the mice were hung on a bar by the tail for 6 min and immobility behavior was scored. The *ERK5 icKO* mice did not show earlier onset of immobility (*p* = 0.942)^26^, or increased frequency (*p* = 0.840)^27^ or duration of immobility (*p* = 0.389)^28^ (Fig. 12), signs of increased behavioral despair.

### *ERK5 icKO* mice do not show enhanced behavioral despair after chronic unpredictable stress treatment

**Figure 13:**
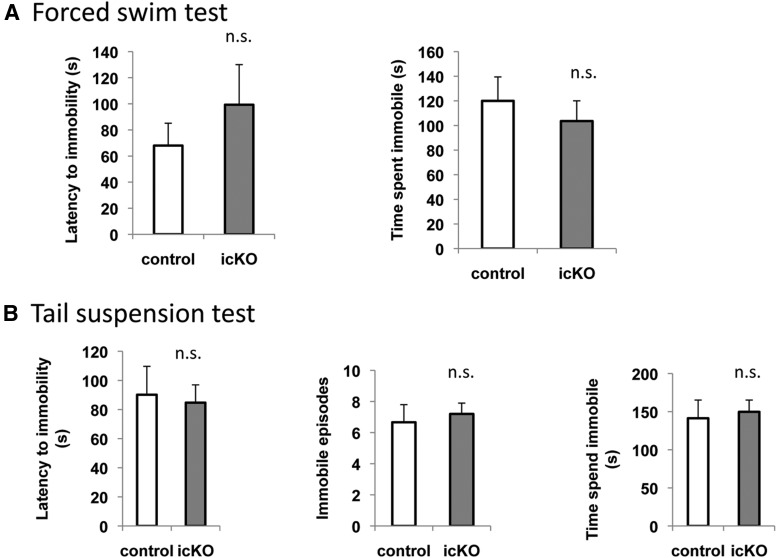
*ERK5 icKO* mice do not exhibit enhanced depression after a 4 week course of CUS treatment. ***A***, Latency to the first episode of immobility from the onset of the test, and the accumulated duration of immobility in the last 4 min of the forced-swim test. ***B***, Latency of immobility, the total number of episodes of immobility, and the accumulated duration of immobility in the tail suspension test. n.s., Not significant.

Finally, we investigated whether impaired adult neurogenesis increases the animal’s susceptibility to depression in the context of chronic stress, using a modified CUS protocol. Stressors included food and water deprivation, cage tilt and wet bedding, cold swim and warm swim, electrical foot shock, tail pinch, and alteration of light/dark cycle with a randomized schedule for 4 weeks. Mice were then subjected to the forced-swim or tail-suspension tests to evaluate depression. There was no difference between *ERK5 icKO* and control mice in their latency to immobility (*p* = 0.389)^29^ and time spent immobile (*p* = 0.527)^30^ in the forced-swim test (Fig. 13*A*), and in their latency to immobility (*p* = 0.816)^31^, frequency of immobility (*p* = 0.694)^32^, and time spent in immobility (*p* = 0.771)^33^ in the tail-suspension test (Fig. 13*B*). These data indicate that the inhibition of adult neurogenesis by ERK5 deletion does not increase the animal’s susceptibility to depression induced by chronic unpredictable stress.

### The same cohort of *ERK5 icKO* mice that have impaired hippocampus-dependent memory do not show anxiety/depression-like behavior

**Figure 14:**
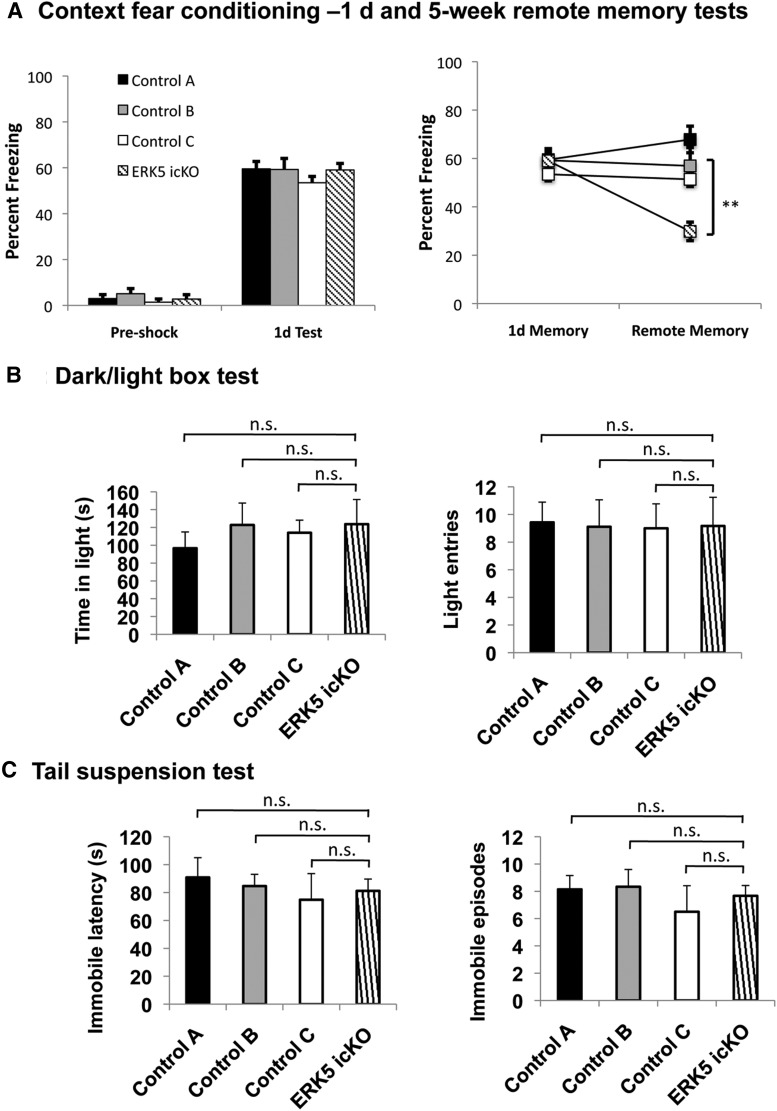
The same cohort of *ERK5 icKO* mice that show impaired hippocampus-dependent memory do not exhibit anxiety/depression-like behavior. ***A***, *ERK5 icKO* mice are impaired in remote memory when the *erk5* gene was inducibly deleted. Data are from Figure 2 of Pan et al. (2012a). **p < 0.01. ***B***, ***C***, These mice are not deficient in a dark/light exploration test (***B***) or tail-suspension test (***C***), performed sequentially after the remote memory test. n.s., Not significant.

The behavior data in Figures 4-13 suggest that impaired adult neurogenesis does not lead to anxiety/depression-like behaviors. Since these data are all negative, we performed another set of experiments using a fourth cohort of *ERK5 icKO* mice, which had been subjected to hippocampus-dependent learning and memory behavior tests. In addition, *ERK5^loxP/loxP^* littermates similarly treated with tamoxifen or vehicle were added as genotype controls. The *ERK5 icKO* mice showed reduced remote contextual fear memory (Pan et al., 2012a) (Fig. 14*A*) (*p* = 0.00055*^34^*, *p* = 0.016*^35^*, and *p* = 0.010*^36^* for percent freezing on a 5 week test, when *ERK5 icKO* mice were compared with control A, B, and C, mice respectively). However, they showed no change in the dark/light box test (Fig. 14*B*) and tail-suspension test (Fig. 14*C*) compared to all three groups of control mice, including genotype controls and tamoxifen drug controls (*p* = 0.436*^37^*, *p* = 0.980*^38^*, and *p* = 0.765*^39^* for time in light, and *p* = 0.920*^40^*, *p* = 0.985*^41^*, and *p* = 0.952*^42^* for light entries in the dark/light box test; *p* = 0.580*^43^*, *p* = 0.796*^44^*, and *p* = 0.769*^45^* for immobile latency, and *p* = 0.714*^46^*, *p* = 0.665*^47^* and *p* = 0.589*^48^* for the immobile episodes in the tail-suspension test when *ERK5 icKO* mice were compared with control A, B, and C mice, respectively).

## Discussion

Because of the tissue- and cell type-specific expression of ERK5; the selectivity, specificity, and sustainability of Nestin-CreER™-driven *erk5* deletion; and a demonstrated role for ERK5 in adult neurogenesis, the *ERK5 icKO* mice provide an excellent animal model to examine a functional role of adult hippocampal neurogenesis in the etiology of anxiety and depression, since other brain regions outside of the adult neurogenic zones are not affected in the *ERK5 icKO* mice. Because an animal’s emotionality and affect are multidimensional, and most of the behavioral tests are based on spontaneous exploration or physical responses and thus involve many nonpsychiatric functions, including locomotion, vision, and hearing, an animal model may be deficient in one aspect of the emotion and mood but not in others (Ramos, 2008). Thus, we performed extensive behavioral assays to assess the level of anxiety and depression-like behaviors in *ERK5 icKO* mice. We found that the *ERK5 icKO* mice are not deficient in any of the nine tests examined, indicating that impaired adult neurogenesis as a result of *erk5* deletion does not alter emotionality and mood.

Numerous studies have reported that intact adult neurogenesis is required for modulating the adaptive responses to chronic stress and for the therapeutic efficacy of antidepressants. However, there is limited evidence for adult neurogenesis in maintaining appropriate affect and emotion in naïve and non-stressed animals (Petrik et al., 2012). For example, cranial X-ray irradiation, which effectively eradicates adult neurogenesis in most studies (Saxe et al., 2006; Clelland et al., 2009; Kitamura et al., 2009), is not associated with anxiety or depression-like phenotypes in naïve or non-stressed animals in the majority of studies using this animal model (Santarelli et al., 2003; Saxe et al., 2006; Surget et al., 2008; David et al., 2009; Surget et al., 2011). The deletion of TrkB in GFAP^+^ neural stem cells, which suppresses hippocampal neurogenesis, is not associated with increased anxiety or depression in non-stressed mice, assessed by the dark/light box and open-field tests or forced-swim and tail-suspension tests, respectively (Li et al., 2008). If anything, these mice are less anxious and spend more time in the open arms of the elevated-plus maze. Thus, there is no clear dose−response correlation between the extent of impairment of adult neurogenesis and the expression of anxiety and depression-like phenotypes. Even more unexpectedly, the reduction of adult neurogenesis by deletion of the cell cycle checkpoint gene *ATR* produces an anxiolytic phenotype in marble burying, novelty-induced hypophagia, and zero-maze tests (Onksen et al., 2011), while enhanced adult neurogenesis by voluntary exercise increases anxiety (Fuss et al., 2010).

Since global approaches such as irradiation to ablate neurogenesis may lead to off-target effects, inducible and cell-specific manipulations, which directly and selectively target adult neurogenesis, have been used to assess whether adult neurogenesis is a regulator of anxiety and depression. The results regarding both the presence and types of phenotypes (anxiety or depression) from a number of these cell-specific transgenic animal models have also been inconsistent. For example, when the pro-apoptotic protein Bax is inducibly expressed in Nestin-expressing adult neural stem cells, inhibition of adult neurogenesis presents an anxiety-like phenotype (elevated-plus maze, dark/light box, and predator-avoidance tests) although it had no effect on depression (forced-swim and novelty-suppressed feeding tests) (Revest et al., 2009). In contrast, when over 99% of doublecortin^+^ newborn neurons are eliminated in the dentate gyrus of transgenic mice expressing thymidine kinase under the control of GFAP promoter upon valganciclovir treatment, it does not alter the baseline level of anxiety (elevated-plus maze), yet causes depression (forced-swim and sucrose-preference tests) (Snyder et al., 2011). Our data indicate that conditional impairment of adult neurogenesis by inducible *erk5* deletion does not lead to anxiety or depression behavior in non-stressed mice.

Whether a deficiency in adult neurogenesis increases an animal’s susceptibility to anxiety and affective disorders in the context of stress is an interesting, open question. When mice were subjected to 6 weeks of CUS and then injected with methylazoxymethanol to impair adult neurogenesis, the mice had exacerbated CUS-induced anxiety-like behavior, indicated by an increased latency in the novelty-suppressed feeding test (Bessa et al., 2009). Snyder et al. (2011) also reported that, after acute stress resulting from being restrained for 30 min, mice lacking adult neurogenesis show increased depression-like behavior in the novelty-suppressed feeding assay. Mice with suppressed adult hippocampal neurogenesis due to the expression of a pathological tau protein in Nestin-expressing neural stem cells show no increase in anxiety in an elevated-plus maze assay in a non-fearful environment (rooms with dim light and no noise), but do show a dramatic reduction of explorative activity in a fearful environment (rooms with bright light and moderate noise) (Pristerà et al., 2013). In contrast, stress resulting from chronic administration of corticosterone does not cause increased anxiety in X-ray-irradiated mice compared with the sham control (David et al., 2009). In addition, several other studies did not observe an effect of impaired adult neurogenesis on CUS-induced anxiety as measured by the open-field assay, novelty-suppressed feeding test, or cookie test (Surget et al., 2008; David et al., 2009; Surget et al., 2011). In the present study, *ERK5 icKO* and control mice performed similarly post-CUS in the forced-swim test and tail-suspension test, indicating that disruption of adult neurogenesis by *erk5* deletion does not increase the animal’s susceptibility to depression after CUS treatment.

It is not clear why different studies using cell-specific manipulations to target adult neurogenesis have led to contradictory conclusions. Some possibilities include differences in the sex, strain and genetic differences of the animals, details of the experimental design of behavior assays, and the efficiency and specificity of the methods used to ablate adult neurogenesis. For example, the mice were in the CD1:C57/Bl6 background, the thymidine kinase expression was driven by the GFAP promoter, and acute restraint was used as a stress model in the study using GFAP-thymidine kinase transgenic mice (Snyder et al., 2011). Our *ERK5 icKO* mice were in the C57/Bl6:129 background, *erk5* deletion was driven by the Nestin-promoter, and mice were subjected to chronic unpredictable stress. Furthermore, expression of thymidine kinase kills 98% of 1-d-old newborn neurons (Snyder et al., 2011), whereas *erk5* deletion in our *ERK5 icKO* mice impairs adult neurogenesis by inhibiting neuronal differentiation but does not reduce the total number of surviving BrdU^+^ cells (Pan et al., 2012c; Pan et al., 2012d). It is conceivable that cell death triggered by transgenic expression of a lethal gene, such as thymidine kinase or the pro-apoptotic protein Bax, exceeds normal physiological level, thus causing effects that are not observed by deleting a specific endogenous signaling molecule like ERK5. Indeed, similar to the report here, two other studies also used Nestin-Cre-ER technology to delete TrkB or fragile X mental retardation protein (FMRP) and found that impaired adult hippocampal neurogenesis by conditional deletion of these endogenous signaling molecules is not associated with depression (assessed by the novelty-suppressed feeding and tail-suspension test, respectively) (Li et al., 2008) or anxiety-like behaviors (elevated-plus maze test) in non-stressed mice (Guo et al., 2011)). Alternatively, anxiety/depression is not very sensitive to adult neurogenesis and only manifests when extensive loss of adult-born neurons occurs.

It is interesting that conditional *erk5* deletion impairs hippocampal adult neurogenesis and hippocampus-dependent memory formation, but has no effect on anxiety/depression. Several other mouse models also have disrupted learning and memory upon impairment of adult neurogenesis but do not show depression- or anxiety-like effects. For example, Saxe et al. (2006) reported that although X-ray-irradiation impaired some forms of hippocampus-dependent learning and memory, it did not affect animal behaviors in the elevated-plus maze or dark/light box tests. In the study by Guo et al. (2011), which is most analogous to our report here, Nestin-cre-ER-driven conditional knockout of FMRP reduced hippocampal neurogenesis and impaired hippocampus-dependent learning and memory. However, there was no change in animal behavior in the elevated-plus maze test (Guo et al., 2011, their Supplemental Fig. 8). It is possible that cell-stage-specific properties of adult-born neurons contribute to learning and memory versus anxiety/depression. For example, immature, adult-born neurons have higher sensitivity to lower threshold stimulation and are more likely activated by weak afferent activity than mature neurons in the dentate gyrus (Marín-Burgin et al., 2012). Thus, adult-born neurons may be uniquely suited for new synapse recruitment during memory formation. In contrast, the total number of neurons in the hippocampal circuitry may be important for total synaptic strength. Since adult neurogenesis only contributes to a very small population of neurons in the hippocampus, reduced adult neurogenesis in many of the animal models, although interfering with memory formation, may not be sufficient to cause synaptic depression and depressive behavior.

Together, our data from the *ERK5 icKO* mouse model indicate that inhibition of adult neurogenesis is not associated with anxiety and depression-like behaviors in non-stressed mice, nor does it increase an animal’s susceptibility to depression after chronic unpredictable stress. Our findings support the notion that intact adult neurogenesis is not essential for maintaining appropriate emotion and affect.

## Synthesis

The decision was a result of the Reviewing Editor Michael Sofroniew and the peer reviewers coming together and discussing their recommendations until a consensus was reached. A fact-based synthesis statement explaining their decision and outlining what is needed to prepare a revision is listed below. The following reviewer agreed to reveal his identity: Francis Szele

### Reviewer #1

The paper is substantially improved and the large majority of my concerns have been addressed. In particular the specificity of the Erk5 loss in the DG (and SVZ) is now clarified and importantly shown in the new Figs. 2-3. Also a positive behavioural control has been added in the new Fig. 14. The new Fig. 1 is very helpful in terms of the timing of the treatments and the behavioural tests carried out. The discussion is significantly improved.

### Reviewer #2

The experimental designs are reasonable, and the manuscript was well written. The revision adequately addressed the reviewers' concerns.
